# Diffuse flow environments within basalt- and sediment-based hydrothermal vent ecosystems harbor specialized microbial communities

**DOI:** 10.3389/fmicb.2013.00182

**Published:** 2013-07-24

**Authors:** Barbara J. Campbell, Shawn W. Polson, Lisa Zeigler Allen, Shannon J. Williamson, Charles K. Lee, K. Eric Wommack, S. Craig Cary

**Affiliations:** ^1^Department of Biological Sciences, Life Science Facility, Clemson UniversityClemson, SC, USA; ^2^Delaware Biotechnology Institute, University of DelawareNewark, DE, USA; ^3^J. Craig Venter InstituteSan Diego, CA, USA; ^4^Lake Pend Oreille WaterkeeperSandpoint, ID, USA; ^5^Department of Biological Sciences, University of WaikatoHamilton, New Zealand

**Keywords:** diffuse flow, microbial diversity, 16S rRNA, pyrosequencing, hydrothermal vents

## Abstract

Hydrothermal vents differ both in surface input and subsurface geochemistry. The effects of these differences on their microbial communities are not clear. Here, we investigated both alpha and beta diversity of diffuse flow-associated microbial communities emanating from vents at a basalt-based hydrothermal system along the East Pacific Rise (EPR) and a sediment-based hydrothermal system, Guaymas Basin. Both Bacteria and Archaea were targeted using high throughput 16S rRNA gene pyrosequencing analyses. A unique aspect of this study was the use of a universal set of 16S rRNA gene primers to characterize total and diffuse flow-specific microbial communities from varied deep-sea hydrothermal environments. Both surrounding seawater and diffuse flow water samples contained large numbers of Marine Group I (MGI) *Thaumarchaea* and *Gammaproteobacteria* taxa previously observed in deep-sea systems. However, these taxa were geographically distinct and segregated according to type of spreading center. Diffuse flow microbial community profiles were highly differentiated. In particular, EPR dominant diffuse flow taxa were most closely associated with chemolithoautotrophs, and off axis water was dominated by heterotrophic-related taxa, whereas the opposite was true for Guaymas Basin. The diversity and richness of diffuse flow-specific microbial communities were strongly correlated to the relative abundance of *Epsilonproteobacteria*, proximity to macrofauna, and hydrothermal system type. Archaeal diversity was higher than or equivalent to bacterial diversity in about one third of the samples. Most diffuse flow-specific communities were dominated by OTUs associated with *Epsilonproteobacteria*, but many of the Guaymas Basin diffuse flow samples were dominated by either OTUs within the *Planctomycetes* or hyperthermophilic Archaea. This study emphasizes the unique microbial communities associated with geochemically and geographically distinct hydrothermal diffuse flow environments.

## Introduction

A defining characteristic of deep-sea hydrothermal environments is that microbial chemosynthetic processes are the primary driver of ecosystem productivity. Thus, a better comprehension of the factors influencing the composition and diversity of vent microbial communities has direct implications for understanding the resilience and productivity of these extreme environments. Previous investigations have found that the taxonomic diversity of hydrothermal vent microbial communities is extensive, particularly when assessed by high throughput sequencing (HTS) approaches (Huber et al., 2007, 2010). There is substantial evidence from standard 16S rRNA gene library and functional gene analyses, as well as metagenomic data that support this conclusion, especially within the *Epsilonproteobacteria* class (Moyer et al., 1995; Lopez-Garcia et al., 2002; Campbell and Cary, 2004; Grzymski et al., 2008; Robidart et al., 2008; Campbell et al., 2009; Nunoura et al., 2010). Archaeal communities at hydrothermal vents are generally thought to be less diverse than coexisting bacterial communities (Huber et al., 2002, 2010; Opatkiewicz et al., 2009; Nunoura et al., 2010). However, these assessments of microbial diversity relied upon PCR primers specific for each domain and are therefore difficult to compare.

The composition of hydrothermal vent-associated microbial communities tends to segregate by vent and distance from actively venting structures (Huber et al., 2007; Opatkiewicz et al., 2009; Dick and Tebo, 2010; Kato et al., 2010; Nunoura et al., 2010). While most of these studies examined either plume water or bottom water, few specifically looked at diffuse flow waters (Huber et al., 2003; Sogin et al., 2006). The factors shaping microbial community diversity and composition are not well understood in these environments. Segregation according to differences in geochemistry is especially prevalent among members of the typically dominant vent bacterial class, *Epsilonproteobacteria* (Nakagawa et al., 2005; Nakagawa and Takai, 2008; Opatkiewicz et al., 2009; Kato et al., 2010). Yet, location seemed to dictate microbial community structure more than geochemistry in other studies (Opatkiewicz et al., 2009; Huber et al., 2010). In fact, recent work showed that endemism was a major factor shaping vent microbial communities even though geochemistry had changed during the 6-year study of the Axial Seamount caldera (Opatkiewicz et al., 2009).

*Epsilonproteobacteria* are a diverse class of mesophilic to moderately thermophilic bacteria that dominate culture-independent surveys of most moderate to high temperature marine hydrothermal vent surfaces. The presence of this class seems to be restricted to associations with macrofauna and the outer surfaces of active vents (Campbell et al., 2006). They are also found in high abundance in some diffuse flow water and plume environments, but were shown to be in low abundance within Guaymas Basin plume 16S rRNA gene libraries (Sunamura et al., 2004; Nakagawa et al., 2005; Dick and Tebo, 2010; Huber et al., 2010). The success of *Epsilonproteobacteria* at hydrothermal vents is likely due to their chemoautotrophic strategy via the reductive tricarboxylic acid (rTCA) cycle along with a moderately versatile metabolism (Campbell et al., 2006). Most vent *Epsilonproteobacteria* are microaerophilic to facultative anaerobes and have the ability to use many sulfur species or hydrogen for energy (Campbell et al., 2006), electron donors found in large quantities at most active deep-sea hydrothermal systems (Von Damm, 1990).

Many other thermophilic Bacteria and Archaea are found less frequently at hydrothermal vents in culture-independent 16S rRNA gene surveys or by quantitative PCR analyses (Gotz et al., 2002; Wery et al., 2002; Huber et al., 2010; Nunoura et al., 2010). Most of these thermophiles and hyperthermophiles appear to also segregate to specific vents, to higher temperature sediments, or internal chimney habitats (Schrenk et al., 2003; Vetriani et al., 2008; Nunoura et al., 2010). One group of mesophilic Archaea, the Thaumarchaeota (formally known as mesophilic Crenarchaeaota) Marine Group I (MGI) clade, are found in high abundance in deep marine waters and in non-diffuse flow hydrothermal vents (Takai et al., 2004; Agogue et al., 2008; Brochier-Armanet et al., 2008; Dick and Tebo, 2010). Although cultured members of MGI are chemoautotrophic ammonium-oxidizers, some uncultured members may be heterotrophic, especially those found in deep water (Konneke et al., 2005; Agogue et al., 2008).

The primary goal of this study was to examine biogeographic and geochemical effects on microbial community composition at vents within the 9°N East Pacific Rise (EPR) and Guaymas Basin vent fields, two widely divergent deep-sea hydrothermal vent environments. Bacterial and archaeal communities within diffuse flow vent fluids and background seawater were examined using a combination of HTS and universal primers for the 16S rRNA gene. While there have been limited studies on symbiotic microbial communities and microcolonizers at these sites, and one study of a Guaymas Basin vent plume, we know of no microbial community surveys from 9°N EPR or Guaymas Basin diffuse flow samples (Haddad et al., 1995; Reysenbach et al., 2000; McCliment et al., 2006; Dick and Tebo, 2010). Because microbial communities within background deep-sea water were also examined, it was possible to identify microbial taxa specific to the unique diffuse flow environments.

## Materials and methods

### Site descriptions and sample collection

Diffuse flow samples were taken with a large volume water sampler (LVWS, Wommack et al., 2004) from various hydrothermal vent and sediment locations at 9°N, EPR, and the Guaymas Basin (Table 1). The LVWS platform was positioned, and its operation was commenced by the DSV Alvin submersible. Temperature was measured at the mouth of the funnel at the start of each collection using Alvin's high temperature probe and therefore indicated the general temperature of the diffuse flow area collected. In addition, small discrete water (SIPPER) samples were taken for chemical analyses (Di Meo et al., 1999). After the LVWS system was purged of surface seawater, diffuse flow water, entrained with bottom seawater, was pumped (~2000L) *in situ* through a 200 μm Nytex net pre-filter, then serially filtered across a 3.0 μm 293 mm filter then two parallel 0.2 μm 293 mm filters (Supor membrane disc filters, Pall Life Sciences) for ~14–16 h. The first ~120L of 0.2 μm filtrate was collected in three Tedlar gas-impermeable plastic bags housed within Nalgene HDPE boxes during each deployment. The LVWS was allowed to surface after an acoustically triggered removal of dive weights, and sample processing occurred immediately upon platform retrieval (within 2 h of triggering).

**Table 1:** **Site descriptions of 9°N East Pacific Rise (EPR) and Guaymas Basin hydrothermal vent samples**.

**Site name**	**Date**	**Dive #**	**Location**	**Depth (m)**	**Temp. (°C)**	**Near macrofauna**	**Notes**
**EPR**							
Tica	11/12/08	4470	9°N 50.414 104°W 17.499	2515	14–19	Y	Located near Riftia and a Tevnia colony.
Bio-9/P-Vent	11/14/08	4472	9°N 50.17367 104°W 17.46176	2511	17–27	Y	Placed near shimmering water, near a Riftia patch.
Marker 28 (Trick or Treat)	11/15/08	4473	9°N 50.17367 104°W 17.46176	2505	30—50	Y	Placed near crabs, next to shimmering water (370 C) black smoker, placed on top of Tevnia patch behind shimmering water.
V-Vent	11/16/08	4474	9°N 50.169 104°W 17.457	2513	22	N	Placed near a crack at the floor of V-Vent.
**GUAYMAS**							
Rebecca's Roost	11/23/08	4476	27°N 0.6763 111°W 24.4168	1987	30	Y	Placed near a Riftia patch near top, scale worms nearby, shimmering water.
Mark's Crack/Pagoda site	11/24/08	4477	27°N 0.4608 111°W 24.5353	2010	26	N	Located near the diffuse flow through crack in vent, white bacterial mat nearby. Hydrocarbon rich.
Rebecca's Roost	11/25/08	4478	27°N 0.6763 111°W 24.4168	1988	27—35	Y	Same location as Dive 4476.
Southern Site/Area	11/26/08	4479	27°N 0.455 111°W 24.573	1999	36	N	Semi-hard bottom, sort of crust on top of sediment; bacterial mat covering; diffuse flow venting out of a crack.
Theme Park	11/27/08	4480	27°N 0.7039 111°W 24.3176	2014	15—22	N	Orange mat, scraped away to reveal bare sediment, white mat around the orange mat, with yellow splotches on the order of ~10 meters.
Southern Site/Area	11/28/08	4481	27°N 0.455 111°W 24.573	1998	35	N	Directly above a hole in an orange mat. The hole has an outpouring of diffuse flow water. Same location as Dive 4479.

Samples collected at the same site on separate dates are indicated.

Two off-axis water samples were taken at least 200 m off the vent axis (one adjacent to Tica −9°N 50.417, 104°W 17.540—at EPR; one near Southern Site—27°N 01.21, 111°W 24.04—at Guaymas) were collected in 30L Niskin bottles on a Carousel Water Sampler/CTD (SBE32; Sea-Bird Electronics) remotely triggered at a depth of 5–15 m above ocean bottom. Samples were immediately filtered and processed on deck using an identical setup as LVWS samples.

### Sample processing and chemical analyses

Ten ml of unprocessed water collected via the SIPPER apparatus was used for all chemical analyses. Aliquots of the sample were separated for dissolved Fe(II) and Fe(total) [defined as Fe(total) = dissolved Fe(III) + dissolved Fe(II)] and analyzed by colorimetry with a Spectronic 601 (Milton Roy) following the ferrozine method (Stookey, 1970). Total sulfide was measured using a standard methylene blue spectrometric method as described previously (Grassoff et al., 1999). Trace element samples (3 mL) were filtered (0.2 mm cellulose nitrate membrane filters) into acid washed glass vials, acidified with 50 μL of concentrated ultra-pure HNO_3_ acid and stored at 4°C until analysis. These samples were prepared for analysis by diluting 50-fold with ultra-pure 2% HNO_3_ and analyzed using a Perkin Elmer Elan SCIEX DRC II inductively coupled plasma mass spectrometer (ICP-MS). To correct for mass bias and instrument drift, a 2% HNO_3_ blank solution and Marek standards were run periodically. pH was measured on each sample using a Orion D2 meter.

### DNA extraction, amplicon generation and sequencing

Immediately upon reaching the surface, the 0.22 μm membranes were aseptically removed from the filter apparatus, placed into sterile plastic bags, and immersed in DNA extraction buffer containing 1× TE, 50 mM EDTA, and 50 mM EGTA. Filters were flash-frozen in liquid nitrogen, held at −80°C while at sea, and returned on dry ice to the J. Craig Venter Institute, San Diego for DNA extraction and library preparation. Methods for DNA extraction from filters can be found elsewhere (Rusch et al., 2007). Briefly, after thawing, the cells were lysed using SDS/Proteinase K and the lysate purified using one phenol extraction and one phenol/chloroform extraction. The supernatant was precipitated using ethanol and eluted in TE buffer. Environmental DNA (eDNA) was then used as template in the PCR targeting 16S rDNA (2 μl). The primers used to isolate the 16S were TX-9, 5′-GGATTAGAWACCCBGGTAGTC-3′ and 1391R, 5′-GACGGGCRGTGWGTRCA-3′ (Ashby et al., 2007; Walker and Pace, 2007), and the following reaction used for gene amplification: 94°C for 3 min, 35 cycles of 94°C for 30 s, 55°C for 30 s, 72°C for 90 s, and 72°C for 10 min. Libraries were barcoded and sequenced using 454-pyrosequencing. The PCR primers were determined to be universal. The 1391R primer has been utilized in the past as a universal primer (Loy et al., 2007) and calculations with Silva testprobe (http://www.arb-silva.de/search/testprobe) estimate the coverage of Bacteria at 88% and Archaea at 76% with one mismatch allowed. The TX-9 primer is not currently present in probeBase, so we calculated an estimated coverage in RDPII (http://rdp.cme.msu.edu/probematch/search.jsp) using the Probe Match tool (Cole et al., 2007). Based on sequences >1200 nucleotides long and of good quality (a total of 1195961 sequences), with one mismatch, the estimated coverage of Bacteria is 99% and Archaea is 98%.

### Sequence analyses

Approximately one million (932,657) sequences were screened for quality by AmpliconNoise as described in detail elsewhere (Quince et al., 2011). A total of 457,209 sequences (average length of 381 bp) passed initial quality filtering. Sequences were then further screened and analyzed in mothur by dereplication, alignment, filtering, preclustering, and average neighbor clustering analyses, with the remaining 456,943 sequences used in clustering, diversity, and taxonomic analyses (Schloss et al., 2009). SFF files were assigned GenBank SRA Bioproject number PRJNA193540.

Ocean floor seawater is often entrained with the vent diffuse flow water during sampling. To calculate which OTUs were significantly enhanced in vent vs. off-axis water, we used statistical tests as described previously (Campbell et al., 2010, 2011). Briefly, significant shifts in OTU abundance between samples were determined in a pairwise fashion by an independent implementation of the statistics used by the RDP LibCompare tool (Wang et al., 2007) using the methods described by Audic and Claverie (1997) and the standard two-population proportions test (Christensen, 1992). Statistically significant results were considered to have a *P*-value less than 0.01 (*P*-values for two-population proportions test were inferred from the Z critical value). In addition, to be included in the pool of sequences belonging to OTUs enhanced in the vent samples, differences in OTU frequency between vent and off axis waters had to be greater than 2-fold. Individual comparisons were between the EPR vent samples and EPR off-axis water or between Guaymas vent samples and Guaymas off-axis water.

Both alpha and beta diversity estimates from the total and vent-specific sequences were calculated in mothur (Schloss et al., 2009). Briefly, the Sobs (observed richness), Chao I (non-parametric estimator of richness), Good's coverage, invsimpson (Inverse Simpson, richness estimator not affected by sampling effort) and np Shannon (non-parametric Shannon index) calculators were implemented to estimate alpha diversity of each sample. Additionally, phylogenetic distances of the samples based on a phylogenetic tree of the vent specific sequences were calculated with the Clearcut program in mothur (Schloss et al., 2009). The beta diversity, similarities between samples from the entire dataset or just the vent-specific OTUs were calculated based on the theta (Yue and Clayton) similarity coefficient (θ_YC_) (Schloss et al., 2009) at a distance of 0.03.

A representative sequence from each OTU was classified by multiple methods, including: Silva, RDPII and greengenes web alignment and classification tools as well as by BLAST analyses (Desantis et al., 2006; Pruesse et al., 2007; Johnson et al., 2008; Cole et al., 2009). In general, the results from all classification schemes were consistent with each other (data not shown).

### Statistical methods

Nonmetric multidimensional scaling (NMDS) was used to examine the relationship between samples based on a θ_YC_ similarity matrix calculated in mothur (Schloss et al., 2009). Only the vent-specific OTUs were used in the analyses. Individual OTUs, which best correlated with NMDS sample distribution by the method of Pearson were overlaid with a biplot, based on vectors calculated in mothur. Only OTUs that were represented by at least 200 sequences and had r values of at least 0.6 were plotted. In addition, correlations between the sample distributions and relative abundances of OTUs were also measured in mothur. Only factors which had r values of at least 0.2 were plotted. NMDS ordinations were also verified in the vegan package in R, using a Bray-Curtis distance calculation. Environmental factors that best correlated to the OTU data were calculated with the bioenv function in R and mothur after log transformation (Schloss et al., 2009) (http://www.r-project.org/).

### Heat map methods

Relative abundance of each OTU served as input for the R PhyloTemp function (a phylogenetically enabled adaptation of the heatmap.2 function; R gplots package; http://phylotemp.microeco.org) (Polson, 2007). The resulting heat map displays relative abundance of each OTU across the individual libraries, with neighbor joining phylogenetic clustering of OTU representative sequences displayed along the y-axis and hierarchical clustering (Bray-Curtis) of taxonomic relative abundance on the x-axis.

## Results

### Physical and chemical description of the samples

A variety of diffuse flow samples were collected from both hard basalt-based EPR and hydrocarbon-rich, sediment-based Guaymas Basin hydrothermal vent habitats (Table 1) using a modified large volume water sampler deployed on an elevator platform (Wommack et al., 2004). Half of the samples were collected near macrofaunal communities, while the other half were collected near hydrothermal diffuse flow vents or sediment surfaces, many with prominent bacterial mats. There were two sets of similar samples collected from the same site but on different dates: Rebecca's Roost and Southern site, both from Guaymas Basin (Table 1).

In general, most chemical and physical features, including temperature, were either very similar among the vents or no discernable patterns were observed within or between spreading center types (Table 2). However, cobalt, nickel and iron levels were significantly different between the spreading centers (*t*-test, Co and Ni, *p* < 0.05; Fe, *p* < 0.08). Cobalt and iron levels were 10 or 30 times higher in the EPR than Guaymas samples and nickel was 2.5 times higher in the Guaymas than the EPR samples.

**Table 2:**
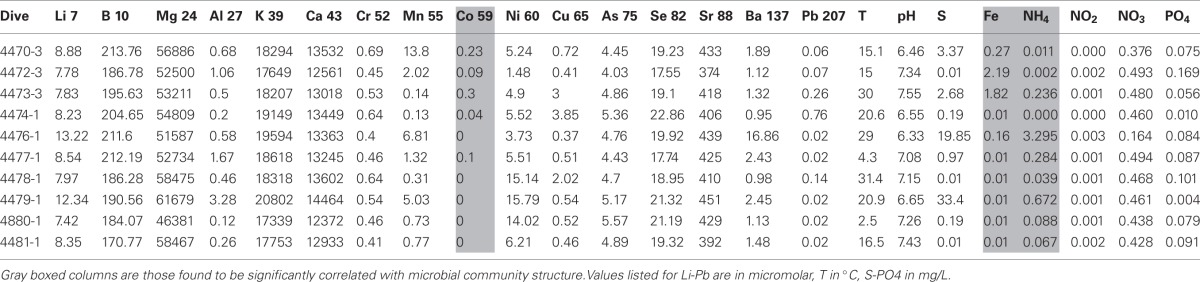
**Chemical characterizations of discrete diffuse flow aquatic water samples**.

Gray boxed columns are those found to be significantly correlated with microbial community structure.Values listed for Li-Pb are in micromolar, T in °C, S-PO4 in mg/L.

### Microbial community diversity

Between 15,000 and 48,000 16S rRNA gene sequences were analyzed from each sample after quality control processing. From the resulting 456,943 sequences, diversity estimates were calculated in mothur (Schloss et al., 2009) and after average neighbor clustering at a 0.03 distance level, 6277 OTUs were produced. The resulting dataset included all archaeal and bacterial sequences because a single set of primers encompassing both domains were used for amplification prior to sequencing (Ashby et al., 2007; Walker and Pace, 2007). The calculated level of Good's coverage from all sequences within each sample was high, between 0.97 and 1 (Table 3). Overall richness estimates (Chao I and Sobs) of the Guaymas Basin sites were much lower than the EPR sites. Conversely, the diversity of the total individual communities as measured by the inverse Simpson or np Shannon calculations did not segregate by geographic location.

**Table 3:** **Diversity estimates from 16S rRNA amplicon libraries of diffuse flow vent samples collected at 9°N East Pacific Rise (EPR) and Guaymas Basin (Guay) hydrothermal vent sites**.

**Sample**	**# seqs^b^**	**Good's coverage**	**S^c^_obs_**	**Chao I richness**	**Simpson evenness**	**Inverse simpson**	**np shannon**
EPR-4470	41,992	0.97	2224	4193	0.0011	2.29	2.68
EPR-4472	42,092	0.98	1696	3319	0.0011	1.92	2.13
EPR-4473	37,602	0.98	1216	2468	0.0032	3.82	3.06
EPR-4474	44,487	0.98	1141	2535	0.0017	1.96	1.85
Guay-4476	48,670	0.99	512	1194	0.0033	1.65	1.12
Guay-4477	29,886	0.99	312	744	0.0069	2.17	1.65
Guay-4478	34,086	0.99	732	1519	0.0028	2.03	1.66
Guay-4479	23,965	0.99	588	1325	0.0054	3.12	2.31
Guay-4480	15,196	0.99	291	690	0.0076	2.19	1.70
Guay-4481	17,152	0.99	336	1102	0.0061	2.03	1.55
EPR OA^a^	76,838	1.00	507	1073	0.0036	1.83	1.40
GUAY OA	44,977	1.00	289	663	0.0114	3.28	2.08

a OA, off-axis.

b #seqs, number of 16S rDNA amplicon sequences.

c Sobs, number of observed species.

**Figure 1:**
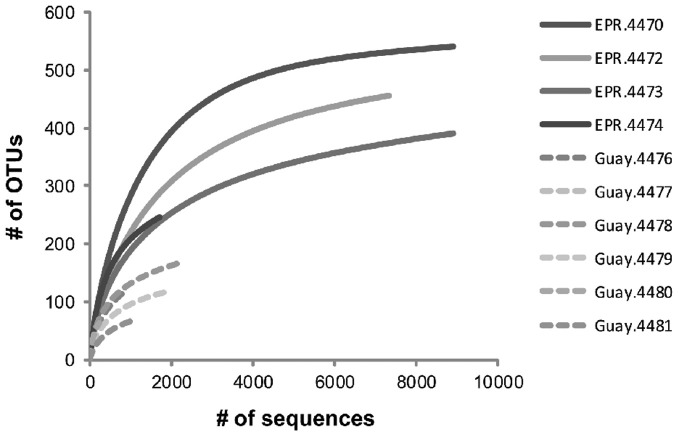
**Rarefaction analysis of vent-specific 16S rDNA amplicon sequences from diffuse flow samples collected at 9°N East Pacific Rise (EPR) and Guaymas Basin (Guay) hydrothermal vent sites**.

To examine the diversity and structure of microbial communities specific to diffuse flow waters, OTUs that were not statistically different between vent and off-axis water were removed from the dataset using previously described statistical methods (Wang et al., 2007; Campbell et al., 2010, 2011). 826 OTUs (at a 0.03 distance) were significantly enriched in vent vs. off-axis water, for a total of 33,001 sequences (about 7% of the total). The number of vent-specific sequences per sample ranged from 0.5 to 25.3% of the total number of sequences per sample (between 167 and 9516 vent specific sequences, Table 4). There were 396 and 90 OTUs that were significantly enriched in the EPR and Guaymas vent samples compared to off axis water, respectively. Of those, about 4% of the EPR and 28% of the Guaymas OTUs were also present in off-axis waters. Generally, vent-specific Guaymas communities displayed lower diversity than EPR communities (Figure 1) even after normalization for differences in sequencing effort (data not shown). Good's coverage estimates were about the same for the EPR communities (0.96–0.99) and only slightly lower for the Guaymas communities (0.85–0.98). Both richness estimates were lower in the vent-specific communities (Table 4), but paralleled the total community richness estimates (Table 3).

**Table 4:** **Diversity estimates from vent-specific 16S rDNA amplicons of diffuse flow vent samples collected at 9°N East Pacific Rise (EPR) and Guaymas Basin (Guay) hydrothermal vent sites**.

**Sample**	**No of seqs**	**% of total^a^**	**Good's coverage**	**Sobs**	**Chao I**	**Simpson even**	**Inv Simpson**	**np shannon**	**Phylo diversity^b^**
EPR-4470	8582	20.4	0.99	538	574	0.0733	39.41	4.93	3.75
EPR-4472	7187	17.1	0.99	452	5084	0.0649	29.32	4.49	3.60
EPR-4473	9516	25.3	0.99	390	484	0.0729	28.42	4.35	2.68
EPR-4474	1667	3.7	0.96	240	315	0.1339	32.14	4.65	5.08
Guay-4476	757	1.6	0.94	114	166	0.1773	20.21	3.84	4.44
Guay-4477	163	0.5	0.87	44	70	0.2195	9.66	3.25	4.60
Guay-4478	2024	5.9	0.98	164	213	0.1683	27.60	4.06	3.47
Guay-4479	1888	7.9	0.98	108	137	0.0328	3.55	2.60	2.96
Guay-4480	164	1.1	0.85	57	80	0.5678	32.36	3.93	5.12
Guay-4481	965	5.6	0.98	59	73	0.0324	1.91	1.65	2.29

a Percentage of total library that were considered as vent-specific sequences.

b Based on phylogenetic distances of 200 sequences (rarefied).

**Figure 2:**
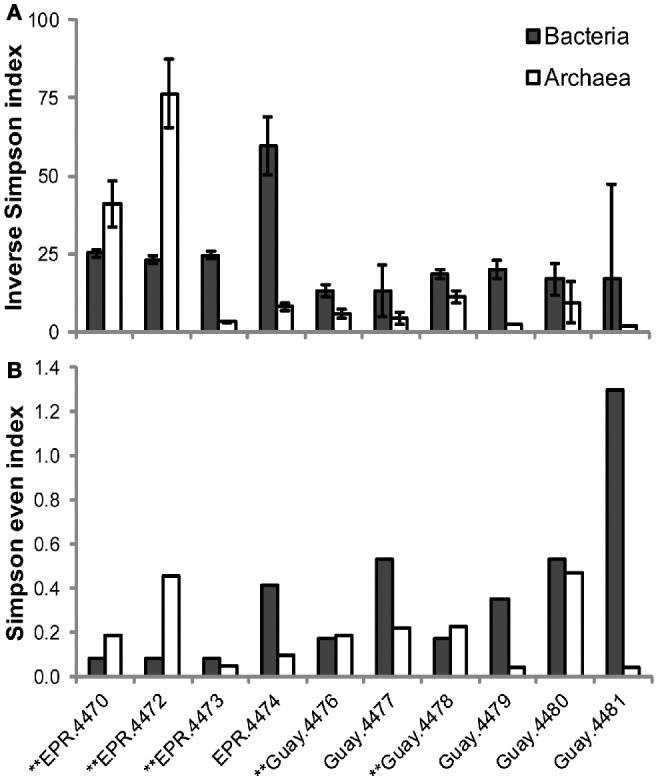
**Differential estimation of archaeal and bacterial diversity (A) and evenness (B) in vent-specific 16S rDNA amplicon sequences from diffuse flow samples collected at 9°N East Pacific Rise (EPR) and Guaymas Basin (Guay) hydrothermal vent sites.** ^**^Indicates macrofauna-associated diffuse flow site.

**Figure 3:**
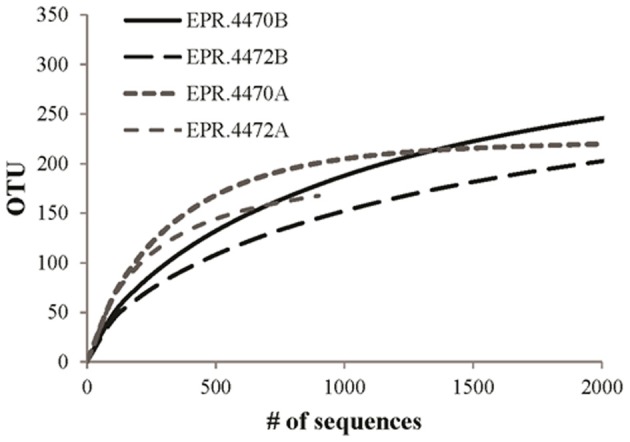
**Rarefaction analysis of archaeal (A) and bacterial (B) vent-specific 16S rDNA amplicon sequences from two 9°N East Pacific Rise (EPR) sites**.

**Figure 4:**
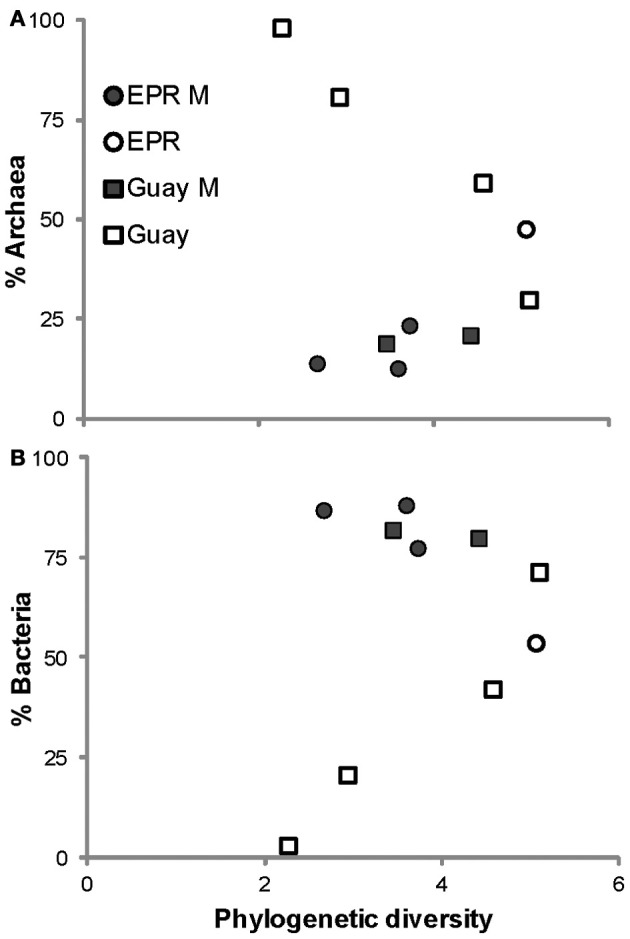
**Relationships between the proportions of Archaea (A) or Bacteria (B) within vent-specific 16S rRNA sequences against estimates of phylogenetic diversity from the indicated sample site.** M, macrofauna-assocated sites, EPR, 9°N East Pacific Rise; Guay, Guaymas Basin.

A direct assessment of differences between bacterial and archaeal diversity from individual samples showed that, in most samples, richness and diversity was higher in Bacteria than Archaea (Table 5, Figures 2, 3). However, in two of the samples (4470 and 4472), archaeal diversity was higher than bacterial diversity and in another sample (4480), they were not statistically different. Archaeal and bacterial evenness was more similar among the vent sites, where three of the samples (4476, 4478, and 4480) had roughly equivalent evenness estimates (Table 3, Figure 2). Interestingly, the two samples with the highest archaeal diversity and richness also had the highest archaeal evenness (4470 and 4472) (Table 5, Figure 2B). Phylogenetic diversity was not correlated with the percentage of Archaea or Bacteria, but the highest indices were found in the communities with roughly equal frequencies of archaeal and bacterial sequences (EPR—4474, Guaymas—4477; Figure 4). Neither sample was located near a macrofaunal community. Samples with low phylogenetic diversity either contained high levels of archaeal sequences and were from non-macrofaunal associated sites, or contained high levels of bacterial sequences and were from macrofaunal associated sites (Figure 4).

**Table 5:** **Diversity estimates from 16S rRNA amplicon libraries of diffuse flow vent samples collected at 9°N East Pacific Rise (EPR) and Guaymas Basin (Guay) hydrothermal vent sites**.

**Group**	**#seqs^a^**	**Sobs^b^**	**Chao I**	**Chao I lci^c^**	**Chao I hci^d^**	**np shannon**	**Inverse simpson**	**Invsimpson lci**	**Invsimpson hci**	**simpson evenness**
**BACTERIA DIFFUSE FLOW VENT SPECIFIC OTUs**										
EPR.4470	6810	305	334	317	377	4.24	25.19	24.05	26.44	0.083
EPR.4472	6380	275	301	287	328	4.00	23.05	22.03	24.16	0.084
EPR.4473	8080	303	356	330	408	4.17	24.39	23.27	25.63	0.081
EPR.4474	867	145	188	165	239	4.55	59.60	52.58	68.78	0.411
Guay.4476	653	77	104	88	145	3.33	13.17	11.74	14.99	0.171
Guay.4477	72	25	41	29	83	3.07	13.24	9.58	21.47	0.530
Guay.4478	1697	105	120	111	148	3.57	18.38	17.06	19.92	0.175
Guay.4479	387	57	78	64	117	3.45	20.03	17.68	23.11	0.351
Guay.4480	143	32	38	34	59	3.18	16.95	13.78	22.02	0.530
Guay.4481	23	13	18	14	41	2.89	16.87	10.26	47.45	1.297
**Group**	**#seqs**	**Sobs**	**Chao I**	**Chao I lci**	**Chao I hci**	**np shannon**	**Inverse simpson**	**Invsimpson lci**	**invsimpson hci**	**simpson evenness**
**ARCHAEA DIFFUSE FLOW VENT SPECIFIC OTUs**										
EPR.4470	2043	220	224	221	236	**4.79^e^**	**41.14**	35.74	48.45	0.187
EPR.4472	908	168	198	181	236	**4.77**	**76.26**	67.78	87.15	0.454
EPR.4473	1289	68	118	86	204	2.19	3.27	3.00	3.58	0.048
EPR.4474	767	86	105	93	136	3.21	8.04	7.02	9.41	0.094
Guay.4476	183	31	51	37	97	2.54	5.73	4.74	7.24	0.185
Guay.4477	102	19	31	22	73	2.21	4.18	3.19	6.06	0.220
Guay.4478	416	49	73	57	124	3.04	11.23	9.81	13.15	0.229
Guay.4479	1553	55	63	57	86	1.85	2.42	2.26	2.60	0.044
Guay.4480	60	20	38	24	95	2.80	9.37	6.62	15.97	0.468
Guay.4481	953	49	56	51	75	1.55	1.87	1.74	2.02	0.038

Sequences derived after subtraction of off-axis OTUs.

a #seqs, number of 16S rDNA amplicon sequences.

b Sobs, number of observed species.

c Low confidence interval.

d High confidence interval.

e Bold values indicate sites where estimated diversity of Archaea exceed that of Bacteria.

### Community composition

**Figure 5:**
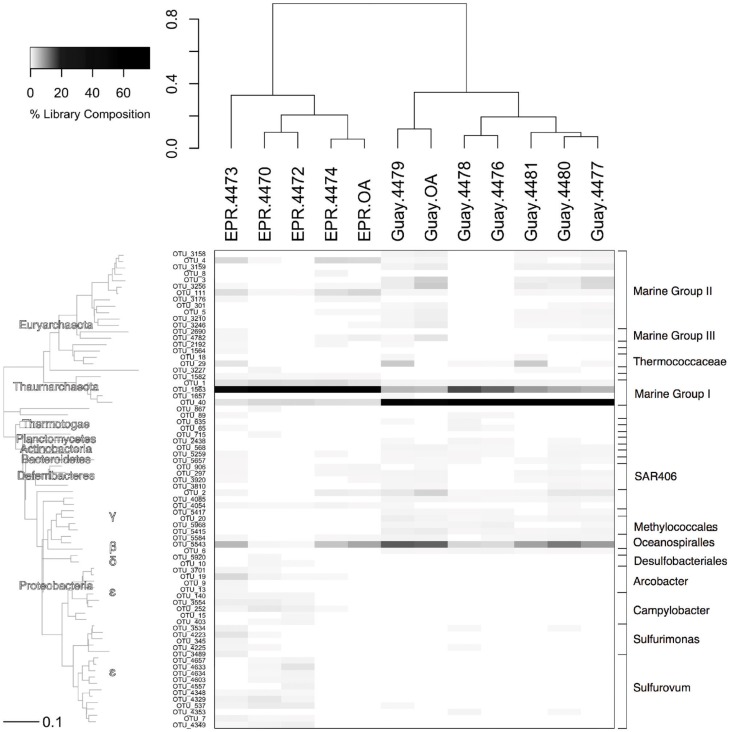
**Relative abundance of OTUs from the entire microbial community of the indicated sample with their corresponding phylogenetic affiliation.** Sample communities were clustered with a Bray-Curtis similarity measurement based on relative abundance data of each OTU. The dendogram on the y-axis is a neighbor joining phylogram derived from representatives from each OTU at abundances greater than 0.25%. The closest related sequences to OTU 1563 were GenBank accession numbers FJ150820 and HM103762, to OTU 40 is HQ331116 and to OTU 5543 is GQ345917.

Microbial community composition of the various samples was assessed by phylogenetic analysis of 16S rRNA gene OTUs present in the entire sample and after subtraction of off-axis OTUs. Overall, the two most abundant OTUs (OTUs 1563 and 40) belonged to the Marine Group 1 (MGI) *Thaumarchaeota*, and their abundance segregated by geographic location where they made up about 60% of the community in either the EPR or Guaymas locations (Figure 5). The closest related sequences to the EPR MGI OTU (0.002 phylogenetic distance) were from North Atlantic deep water and the Sea of Marmara. The Guaymas MGI OTU was more closely related to *Nitrosopumilus* sp. (0.011 distance). The second most abundant OTU belonged to the *Oceanospirillales* (SUP05) within the *Gammaproteobacteria* and comprised about 7 and 13% in the EPR and Guaymas off-axis bacterioplankton, respectively; and between 0.1 and 3% in some of the diffuse flow samples (EPR-4470, 4472, Guay-4476, 4478) (Walsh et al., 2009).

Other phylotypes that were present at 0.5% or greater abundance in both EPR and Guaymas vent and off axis water included members of the Marine Groups II and III (MGII, MGIII) *Euryarchaeota*, as well as *Deltaproteobacteria* and *Deferribacteres* SAR406 (Marine Group A) bacterial clades. OTUs more abundant at Guaymas than EPR included members of the *Methylococcales* and *Thiotrichales* (*Gammaproteobacteria*), *Methylophilales* (*Betaproteobacteria*), and *Desulfobacterales* (*Deltaproteobacteria*) and ranged from about 20- to more than 200-fold more abundant in Guaymas than EPR off-axis waters.Prevalence of many of these taxa, especially the *Methylococcales* and *Methylophilales*, are likely related to the high methane and hydrocarbon concentrations of the Guaymas spreading center (Edmond et al., 1982; Von Damm et al., 1985).

**Figure 6:**
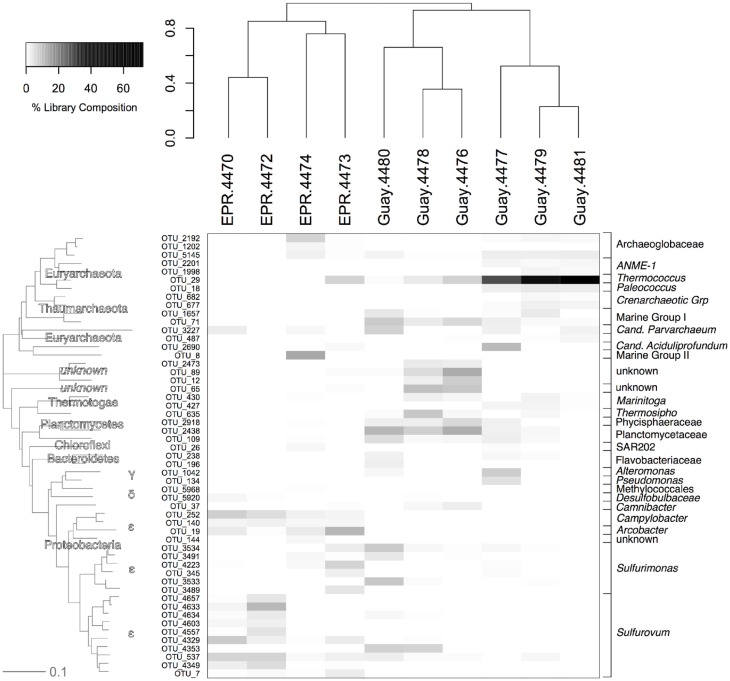
**Relative abundance of OTUs from the vent-specific microbial community of the indicated sample with their corresponding phylogenetic affiliation.** Sample communities were clustered with a Bray-Curtis similarity measurement based on relative abundance data of each OTU. The dendogram on the y-axis is a neighbor joining phylogram derived from representatives from each OTU at abundances greater than 0.25%. EPR, 9°N East Pacific Rise; Guay, Guaymas Basin.

Among OTUs that were significantly enriched or specific to diffuse flow samples, three were found in every diffuse flow sample (Figure 6). The first, an OTU within the *Planctomycetales* (OTU-2438), with a range of frequency between 0.01 and 15%, was more prevalent in the Guaymas than EPR diffuse flow samples. The second, a member of the Deep Sea Hydrothermal Vent Group 6 archaeal clade (OTU-3227), ranged in abundance from 0.01 to about 10% and did not segregate by geographic location. The last OTU present in all vent samples, an *Archaeoglobales* (OTU-5145), comprised 0.07–4.3% of the community and was not found at all in off-axis waters. Seven OTUs were found in at least 80% of diffuse flow samples, two of which were within the *Planctomycetes* phylum (OTU-2438 and 2918) and were more prevalent in Guaymas Basin samples than EPR samples. The closest BLAST hits to these *Planctomycetes* were uncultured members of the phylum; all cultured *Planctomycetes* and members of the annamox clade were at least 15% divergent within the amplified region, illustrating the diversity of this group according to 16S rRNA gene sequence (data not shown). One OTU (29), belonging to the *Thermococcales* family, was found in high abundance (3–76% of vent specific sequences) in EPR-4473 and four Guaymas samples (4477, 4478, 4479, and 4481). The other four OTUs (537, 3491, 3534, 4223) belonged to the *Epsilonproteobacteria* and each OTU comprised up to 7% of the vent-specific OTUs in at least 80% of the samples. Of the most abundant vent-specific OTUs, there were eight that were unique to one or two of the diffuse flow samples, but comprised up to 16% of the vent-specific OTUs. One of these was a member of MGII and was not found in off-axis water but only in EPR-4474 (OTU-8). Two other OTUs (144 and 3489) were found in one or two EPR diffuse flow samples respectively and were members of the *Epsilonproteobacteria*. Another OTU (1998), classified as a member of the ANME-1 group, was found only in Guaymas 4479 and 4481. Other Guaymas Basin-specific OTUs (196, 1042) include members of the *Flavobacterales* and *Alteromonadales* families, as well as an unclassified bacterial phylotype (OTU-12).

Half of the sample locations (three of four sites at EPR and two of six sites at Guaymas) were adjacent to significant concentrations/colonies of macrofauna (e.g., Riftia, Tevnia, crabs; Table 1). The diffuse flow samples collected near macrofaunal communities had significantly higher percentages of Bacteria and *Epsilonproteobacteria* than the other samples (*t*-test, *p* < 0.05), mainly driven by *Sulfurovum* (average frequency of 21 vs. 3%). Samples not collected near macrofaunal communities had significantly higher percentages of Archaea than the other samples (*t*-test, *p* < 0.05), with increased frequencies of *Thermococcus* (30 vs. 3%). Despite this, there were no OTUs that were specific to diffuse flow water collected near macrofaunal communities.

We next looked at the archaeal composition of the two samples where the archaeal diversity was significantly higher than the bacterial diversity, EPR-4470 and 4472. EPR-4470 contained 220 different archaeal OTUs, whereas EPR-4472 contained 168. These were much higher than the rest of the samples, where the number of OTUs ranged from 19 to 86 (data not shown). There were 46 OTUs from EPR-4470 that contained 10 or more sequences in each OTU. Less than half that number (19) was in the EPR-4472 sample. There were no dominant OTUs in either sample except for an unclassified Archaea, most likely in the *Methanosphaera* group, found in the EPR-4470 sample. There were more OTUs within the *Euryarchaeota* (54 and 42%, respectively for the EPR-4470 and 4472 samples) than *Crenarchaeota* (20 and 42%) or unclassified Archaea (26 and 16%). In contrast, the samples with the lowest archaeal diversity (Guay-4477 and Guay-4480) were composed of 19 and 20 OTUs, respectively, and were dominated by *Thermococcus* and unclassified Archaea (data not shown).

### Comparative analysis of diffuse flow microbial communities

**Figure 7:**
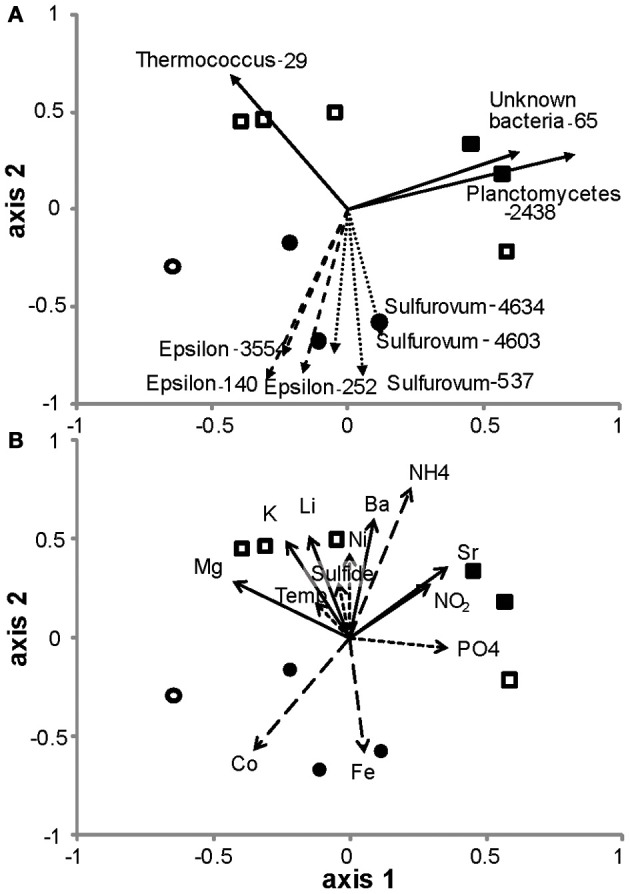
**Non-metric multidimensional scaling plot of hydrothermal vent microbial communities. (A)** Vectors of the correlation values from OTUs that most explain the relationships in the plot (*r* > 0.6, *n* > 2.0). **(B)** Vectors of the correlation values from environmental factors that most explain the relationships in the plot (*r* > 0.2 and <0.4 indicated as dotted lines, *r* > 0.4 and <0.6 indicated as solid lines and *r* > 0.6 as dashed lines). Circles, 9°N East Pacific Rise; squares, Guaymas Basin; closed symbols, macrofauna-associated; open symbols, not macrofauna-associated.

Analysis of diffuse flow communities separated EPR from the Guaymas sites according to both Bray-Curtis similarity (Figure 6) and nonmetric dimensional scaling (NMDS) of the nonparametric Theta – Yue and Clayton (θyc) similarity coefficients (Schloss and Handelsman, 2005; Schloss et al., 2009) (Figure 7). To explain the grouping of the samples along the axes, the correlation of the relative abundance of individual OTUs in the NMDS dataset was calculated. The vector values of the most abundant OTUs were overlaid on the NMDS plot (Figure 7A). The OTUs that most contributed to the spatial distribution of the samples were within the *Epsilonproteobacteria*. About half of these were within the *Sulfurovum* genera, the others did not classify at genera level. Three other OTUs within the *Sulfurovum* genera also were correlated but were left off the plot because of their similarity to the vectors of the other *Sulfurovum* genera (data not shown). A *Thermococcus* (OTU-29) that was found in very high abundance in three Guaymas samples (4477, 4479, 4481), and a *Planctomycetes* (OTU-2918 & 2438) and an unclassified bacteria (OTU-65) found in high abundance in Guaymas 4476, 4478, or 4480 also significantly contributed to the observed distribution (Figure 7A).

Out of 24 constituent geochemical features (Table 2), iron, cobalt and ammonium concentrations correlated best to the community similarity plot (*r* > 0.6, confirmed with function bioenv in *R* where *r* = 0.6) (Figure 7B). Ammonium was correlated more with the distribution of microbial communities in the Guaymas Basin samples, while iron and cobalt were better correlated with communities in the EPR samples. Also included on the plots were constituents whose r values were between 0.2 and 0.6 (Figure 7B). These included temperature, sulfide, nickel, and phosphate (between 0.2 and 0.4) and magnesium, potassium, lithium, barium, strontium, and nitrite (between 0.4 and 0.6).

## Discussion

Our experimental approach allowed us to deeply sample microbial diversity and community composition between two geochemically and geographically distinct hydrothermal vent diffuse flow environments. Using statistical subtraction of taxa found in surrounding deep-sea water, it was possible to directly compare vent-specific taxa and further partition this diversity by taxonomic domain. As with other microbial diversity studies of hydrothermal vent microbial communities (Huber et al., 2007, 2010), *Epsilonproteobacteria* dominated the vent specific taxa in most samples. Samples collected near macrofaunal communities had a higher abundance of bacteria, specifically *Epsilonproteobacteria*, than those collected near sediments or vents not populated by macrofauna. Unexpectedly, we found that some diffuse flow environments contained archaeal communities that were of higher diversity and evenness than co-existing bacterial communities. Additionally, one of the dominant taxa groups found in the off axis water, Thaumarcheaota (formerly marine Crenarchaeota) MGI (Delong, 1992; Brochier-Armanet et al., 2008), was differentially present between the two geographic locations. The second, SUP05, is found in many oxygen minimum zones, including hydrothermal vent plumes (Sunamura et al., 2004; Walsh et al., 2009). Nevertheless, distinct patterns in microbial community composition between sites were apparent after statistical subtraction of taxa present in background seawater. These patterns were correlated both to specific taxa (e.g., *Epsilonproteobacteria*, *Planctomycetes*) and to environmental factors (e.g., iron, ammonium).

### Distinctions in microbial diversity between diffuse flow samples

The diversity of hydrothermal vent microbial communities is extensive, especially when measured with HTS techniques (Huber et al., 2007, 2010). In this study, total microbial diversity in both locations was positively correlated with the prevalence of Bacteria, especially *Epsilonproteobacteria*, within the vent-specific component of the community. Additionally, epsilonproteobacterial abundance as measured by frequency analyses was also significantly correlated with overall microbial richness. Other studies have noted high levels of epsilonproteobacterial diversity in hydrothermal vent environments (Huber et al., 2007, 2010; Opatkiewicz et al., 2009).

Collection near macrofaunal communities was correlated with increased overall diversity but decreased phylum-level diversity. These communities were dominated by *Sulfurovum* spp. within the *Epsilonproteobacteria*. This class of Proteobacteria may be more diverse than other groups due to their genetic makeup, where much of the group lacks standard DNA repair gene pathways seen in other bacterial groups (Miller et al., 2007; Campbell et al., 2009). We also observed higher levels of archaeal than microbial diversity or evenness in three out of five of the macrofauna-associated sites. This finding contrasts with prior reports, where the diversity of archaeal populations was lower than bacterial diversity when different sets of primers and different library constructs are used (Huber et al., 2002, 2003, 2010; Opatkiewicz et al., 2009). The resolution afforded by using a single set of domain-independent primers, as well as sampling of macrofauna-associated and non-macrofauna sites, likely contributed to our discovery of high levels of archaeal diversity at the vent sites.

While diversity estimates were not significantly different for vent-specific microbial communities between geographic locations, which agrees with our Good's coverage estimates, richness estimates were significantly different, even when corrected for sequencing effort. Several location-specific factors may account for the decreased richness observed in the Guaymas samples. Historically, Guaymas Basin hydrothermal fluids are depleted in sulfides and enriched in ammonium, nickel, methane and hydrocarbons as compared to the EPR spreading center (Edmond et al., 1982; Von Damm et al., 1985). Extreme or disturbed conditions often result in less richness (Campbell et al., 2010; Fierer and Lennon, 2011), and these features of the Guaymas site may act to decrease richness. Additional properties not investigated here, such as diffuse flow rates or numbers of particles may also be different between the sites and affect microbial richness. Nevertheless, even at the gross level of richness estimates, there were clear biogeographic effects on deep-sea hydrothermal vent communities.

### Microbial composition analyses of diffuse flow samples

Off-axis microbial communities from each region, EPR and Guaymas, were significantly different from one another. Our analyses revealed that archaeal phylotypes, specifically within the MGI clade, were the most abundant phylotypes in the seawater surrounding hydrothermal vents and differentiated the two regions. The MGI clade has been reported to dominate seawater microbial communities adjacent to hydrothermal vents and plumes, in some cases up to 46% of the entire community (Huber et al., 2002; Takai et al., 2004; Dick and Tebo, 2010). The dominant MGI Thaumarchaeaota OTU at the EPR site grouped with other MGI species within the sub-tropical and equatorial deep water cluster; whereas the dominant MGI OTU at the Guaymas Basin grouped with North Atlantic clones within the *amo*A archaeal isolates cluster. It is likely that most MGI found in deep waters, including the major MGI at the basalt-dominated EPR are different than the MGI at Guaymas in terms of their metabolic properties (Agogue et al., 2008; Bouskill et al., 2012). Despite the low latitude location of the Guaymas site, the occurrence of a taxon related to *amo*A Archaea from North Atlantic suggests that the most abundant MGI species in Guaymas sedimentary-dominated deep-sea water is an autotrophic archaeal ammonium oxidizer. The generally higher ammonium concentrations in the Guaymas samples support this hypothesis. Therefore, our data indicate that the dominant deep-sea taxa from the EPR are most likely heterotrophs or mixotrophs and the dominant taxa from Guaymas are most likely autotrophs. This could be driven mostly by environmental conditions, where Guaymas Basin sites have high levels of ammonium resulting from high temperature breakdown of photosynthetic organisms sinking from the productive surface waters of the Gulf of California (Von Damm et al., 1985). Recent metagenomic and metatranscriptomic studies of both background and plume waters indicated high levels of chemolithoautotrophic processes from Guaymas Basin environment, supporting this findings (Baker et al., 2012; Lesniewski et al., 2012).

After statistical subtraction of OTUs within background deep-sea water, detailed patterns in microbial community structure emerged between the samples from various diffuse flow environments. In general, bacterial species distributions between the EPR and Guaymas indicated a trend toward dominance of autotrophic-associated taxa (*Epsilonproteobacteria*) at the EPR sites and heterotrophic-associated taxa (*Planctomycetes, Alteromonas, Thermosipho, Thermococcus*) at the Guaymas sites. At some of the sites (4472, 4478, 4480), the *Epsilonproteobacteria* were dominated by OTUs related to the *Sulfurovum*, *Sulfurocurvum*, and *Sulfuromonas* genera. Members of these genera are autotrophs and generally mesophilic and microaerophilic, but may respire nitrate, and use various sulfur species as electron donors (Campbell et al., 2006). These sites generally had higher pH and lower sulfide concentrations than others, perhaps indicating that these bacterial groups were actively oxidizing the sulfide. The epsilonproteobacterial communities at the other EPR sites (4470, 4473, 4474) were more evenly distributed between OTUs within the *Sulfurocurvum*, *Sulfuromonas*, and *Sulfurovum* genera and *Arcobacter*/*Sulfurospirillum* genera. While isolates within the *Sulfurospirillum* are heterotrophic, members of the *Arcobacter* can be autotrophic as well, and both can use sulfur as an electron donor (Campbell et al., 2006). Two of the samples taken at the same site (4476 and 4478) had relatively high levels of OTUs within the *Nautiliaceae*. Members of this family are thermophilic anaerobic autotrophs who obtain energy from hydrogen and may respire nitrate (Alain et al., 2002; Voordeckers et al., 2005; Campbell et al., 2006, 2009). Differential dominance of epsilonproteobacterial genera at vents has been previously observed at the Axial and Mariana Arc seamounts (Huber et al., 2007, 2010; Opatkiewicz et al., 2009) but not in the EPR or Guaymas Basin spreading centers.

At most of the Guaymas sites, member genera within the *Planctomycetes* were particularly frequent in the vent-specific samples. The *Planctomycetes* have been described in multiple habitats and include some genera that perform anaerobic ammonium oxidation (Neef et al., 1998; Jetten et al., 2001; Chistoserdova et al., 2004). This is the first study to demonstrate that members of the *Planctomycetes* occur at high abundance (>5% in the vent-specific community) within hydrothermal vent environments, although they have been found in terrestrial thermal springs (Kanokratana et al., 2004; Elshahed et al., 2007). *Planctomycetes* are generally considered heterotrophic, and isolates from low temperature sulfide springs are able to reduce sulfur species, therefore this group may be important in the heterotrophic cycling of sulfur in marine hydrothermal environments as well (Elshahed et al., 2007).

The two Guaymas samples taken from the Southern Site (4479 and 4481), as well as one other Guaymas sample from Pagoda and one of the EPR samples (V-vent) had more than 25% archaeal phylotypes. The high percentage of Archaea in the Guaymas samples was driven by a single OTU belonging to the *Thermococcales* order. Members of the *Thermococcales*, frequently isolated from hydrothermal vents, are generally considered anaerobic heterotrophic hyperthermophiles (Holden et al., 2001; Slobodkin et al., 2001; Jolivet et al., 2004; Teske et al., 2009; Perevalova et al., 2011). V-vent contained a large percentage of members of the *Archaeoglobales* and unclassified Euryarchaeota, many of which may be heterotrophic (Kletzin et al., 2004; Rusch and Amend, 2008). The sulfate-reducing *Archaeloglobales, Theromococcales*, and other Euryarchaeota are most likely hyperthermophiles as well (Stetter, 1996) and thus, diffuse flow from these sites are enriched with microbes originating from very high temperature environments.

While we base our discussion on the known thermal and metabolic properties of cultured Bacteria and Archaea, we did find some interesting trends in the data that suggest that, after subtraction of off-axis microbes, some sites are dominated by thermophilic to hyperthermophilic heterotrophs and others by mesophilic autotrophs. This indicates that not all diffuse flow environments are equally represented by microbes across all thermal regimes. It could also be the result of a lack of complete diversity and compositional coverage of all the samples. However, Good's estimates of coverage were quite high, even with subtraction of OTUs specific to off-axis bottom water.

## Conclusions

This study underscores the utility of using HTS techniques combined with simultaneous amplification of the 16S rRNA gene from both Bacteria and Archaea to statistically separate off axis from vent-specific taxa. We found that overall diversity was significantly lower in the sedimentary Guaymas vent environments than in the basaltic EPR deep-sea hydrothermal vent environments, which has implications for understanding environmental controls of microbial diversity in these extreme habitats (Huber et al., 2007; Quince et al., 2008). Our study also suggests that environment-specific factors such as proximity to macrofaunal communities can have a dramatic effect on microbial community richness, diversity and composition. Furthermore, we observed unique microbial sub-communities that were specialized to the diffuse flow environment and were different between spreading centers. Regrettably, we can only speculate as to the physiological features of member populations within diffuse flow communities as these observations are based on 16S rRNA gene homologies to known microbes. Deeper understanding of connections between phylogeny and physiology in these diffuse flow specialized microbial phyla can only come through genome level studies (Baker et al., 2012; Lesniewski et al., 2012).

### Conflict of interest statement

The authors declare that the research was conducted in the absence of any commercial or financial relationships that could be construed as a potential conflict of interest.
